# Discovery of Potential Inhibitors of Aldosterone Synthase from Chinese Herbs Using Pharmacophore Modeling, Molecular Docking, and Molecular Dynamics Simulation Studies

**DOI:** 10.1155/2016/4182595

**Published:** 2016-10-03

**Authors:** Ganggang Luo, Fang Lu, Liansheng Qiao, Xi Chen, Gongyu Li, Yanling Zhang

**Affiliations:** Beijing Key Laboratory of TCM Foundation and New Drug Research, School of Chinese Material Medica, Beijing University of Chinese Medicine, Beijing 100102, China

## Abstract

Aldosterone synthase (CYP11B2) is a key enzyme for the biosynthesis of aldosterone, which plays a significant role for the regulation of blood pressure. Excess aldosterone can cause the dysregulation of the renin-angiotensin-aldosterone system (RAAS) and lead to hypertension. Therefore, research and development of CYP11B2 inhibitor are regarded as a novel approach for the treatment of hypertension. In this study, the pharmacophore models of CYP11B2 inhibitors were generated and the optimal model was used to identify potential CYP11B2 inhibitors from the Traditional Chinese Medicine Database (TCMD, Version 2009). The hits were further refined by molecular docking and the interactions between compounds and CYP11B2 were analyzed. Compounds with high Fitvalue, high docking score, and expected interactions with key residues were selected as potential CYP11B2 inhibitors. Two most promising compounds, ethyl caffeate and labiatenic acid, with high Fitvalue and docking score were reserved for molecular dynamics (MD) study. All of them have stability of ligand binding which suggested that they might perform the inhibitory effect on CYP11B2. This study provided candidates for novel drug-like CYP11B2 inhibitors by molecular simulation methods for the hypertension treatment.

## 1. Introduction

Cardiovascular diseases (CVDs) are the leading cause of mortality worldwide, including coronary artery diseases (CAD), hypertensive, heart disease, stroke, cardiomyopathy, endocarditis, heart arrhythmia, aortic aneurysms, and peripheral artery disease [1, 2]. Among these diseases, hypertension is a high-incidence cardiovascular disease around the world, leading to 7 million deaths each year and about 25% of adults suffer from the disease [3]. Aldosteronism is one of the primary causes for hypertension [4]. Aldosterone, the main mineralocorticoid hormone, is part of the renin-angiotensin-aldosterone system (RAAS) [5], which plays a significant role in the regulation of blood pressure by increasing blood pressure and blood volume.

Aldosterone synthase (CYP11B2) is a steroid hydroxylase cytochrome P450 enzyme [6], which is the key enzyme responsible for the production of aldosterone in humans. It accelerates the terminal three oxidation steps in synthetic pathway of aldosterone. It is a member of the cytochrome P450 superfamily of enzymes and not only plays an important role in electrolyte balance and blood pressure but also catalyzes many reactions in the regulation of drug metabolism and synthesis of cholesterol, steroids, and other lipids.

CYP11B2 is regarded as promising target for the treatment of hypertension which has gained great attention. CYP11B2 inhibitors are identified using a variety of methods so far. For example, Ulmschneider et al. combined synthesis and biological evaluation methods to obtain pyridylmethylene derivatives as CYP11B2 inhibitors in 2005 [7]. Novel CYP11B2 inhibitors with extended carbocyclic skeleton were obtained by a combined ligand-based and structure-based method [8]. However, the structural frame of CYP11B2 inhibitors does not have structural diversity from chemical synthesis. Herein, more efforts shall be devoted to discover novel CYP11B2 inhibitors from natural products.

The ingredients from Chinese medicine are a new source to obtain candidates with novel chemical structure for treatment of hypertension [9, 10]. For instance, hydroxysafflor yellow A, the primary chemical ingredient of safflower, had been verified with beneficial effects for the treatment of hypertension [11]. In recent years, molecular simulation technologies, including pharmacophore, molecular docking, molecular dynamics (MD), and homologous modeling, have been utilized for new drug research and development. A series of mitochondrial cytochrome P450 superfamily receptors have become hot spots in focus as novel targets to discover potential new drugs. Potential CYP2D6 inhibitors were screened by using pharmacophore, QSAR, and molecular docking methods from Chinese herbal by Mo et al. [12]. Yu et al. combined pharmacophore modeling, 3D-QSAR, homology modeling, and docking to obtain the CYP11B1 inhibitors [13]. With the solution of the crystal structure of CYP11B2, it is worthy of combining molecular simulation technologies to screen potential CYP11B2 inhibitors, analyzing the interactions between compounds and protein, and validating the stability of binding mode.

This study aimed to screen potential CYP11B2 inhibitors from TCMD using molecular simulation methods. Ten HipHop pharmacophore models were generated based on twenty CYP11B2 active inhibitors. The optimal pharmacophore model was selected by the validation of test set and used as a query to search candidates for CYP11B2 inhibitors from TCMD. Then molecular docking was employed to refine the hits of pharmacophore model and analyze the interactions between compounds and receptor. Then MD was performed to examine the stability of compounds and protein. Finally, two compounds were selected as most promising CYP11B2 inhibitors. This study provides a reliable method for discovering CYP11B2 inhibitors from natural products.

## 2. Materials and Methods

### 2.1. Pharmacophore Model Studies

#### 2.1.1. Data Preparation

**Figure 1:**
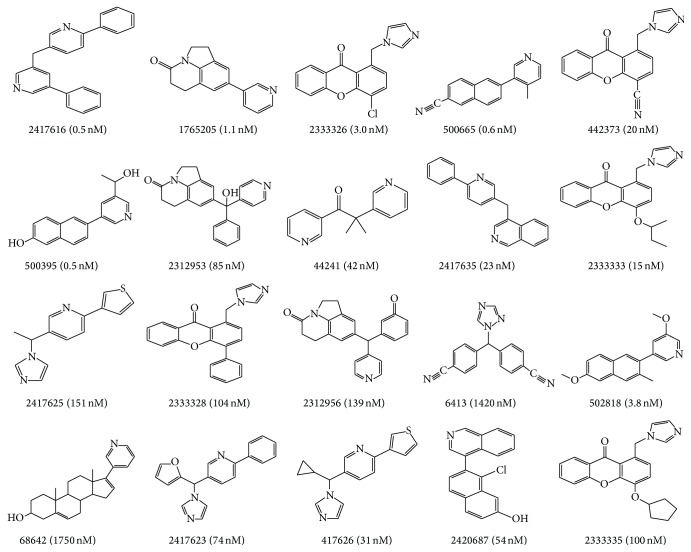
Structures, ID number, and IC_50_ value of twenty active compounds in the training set.

By entering “human CYP11B2 receptor inhibitors” as a search term in the Binding Database (http://www.bindingdb.org/), 91 active compounds were obtained as a data set. Among the compounds, 20 compounds were selected as the training set based on the structural diversity and active value and used to generate HipHop pharmacophore model. Those compounds contained more than four subtypes of chemical structure and the biological activities (IC_50_) ranged from 0.50 nM to 1750 nM. Structures, ID number, and IC_50_ values of compounds in the training set were shown in Figure 1. For validating the pharmacophore model, the test set was built, concluding the remaining 71 active compounds and another 213 inactive compounds were selected randomly from the Binding Database. The conformations of all compounds were generated by the BEST method in Discovery Studio 4.0 (DS 4.0). Each compound generated an energy threshold of 20 kcal/mol and maximum conformations were 255.

#### 2.1.2. HipHop Pharmacophore Model Generation

The HipHop pharmacophore models were built by selecting common pharmacological features among 3D chemical features of the compounds in the training set [14]. A list of pharmacological features was selected for pharmacophore generation, including hydrogen bond acceptor (A), hydrogen bond donor (D), hydrophobic (H), and ring aromatic (R) [15]. During the pharmacophore generation,* Principal *values of compound 2417616, 500395, and 500665 with highest active were set to 2 and corresponding* MaxOmitFeat* values were set to 0. On the contrary, for the two compounds 68642 and 6413 with lowest activity in the training set, the* Principal* values were set to 0 and corresponding* MaxOmitFeat* values were set to 2. Both the* Principal* and* MaxOmitFeat* of the other compounds of the training set were set to 1. The parameters of* FeatureMisses* and* CompleteMisses* were set to 4 and 3. Based on the molecular weight of compounds in the training set, the Minimum Interfeature Distance value was adjusted from 2.97 Å to 0.5 Å to ensure the pharmacological features located closely to each other during pharmacophore generation. The maximum excluded volumes (Ev) value was set to 5; meanwhile, all the other parameters were set at default values.

#### 2.1.3. Validation and Optimization and of Model

The test set was employed to evaluate the predictive power of the generated pharmacophore models. The evaluation indicators were shown as follows: HRA represents the ability to identify active compounds from the test set. IEI represents the ability to identify active compounds from inactive compounds. CAI is the comprehensive appraisal index [16]. ∑Ranking is the sum of the ranking values of the four indicators, containing Rank, HRA, IEI, and CAI. To obtain the highly credible candidates, the study takes the following criterions [15]. (1) The HRA is greater than 80%. (2) The values of IEI and CAI were more than 2. (3) The ∑Ranking value is the lowest. Fadrozole, a known CYP11B2 inhibitor, was also mapped with the best pharmacophore model to validate the accuracy of the optimal pharmacophore model.

To improve the accuracy of pharmacophore model, an optimized process was executed. There are three parameter sets which can be fine-tuned. Firstly, the Tolerance (T) value, which indicates the radius of the sphere of each pharmacodynamic feature, can be manually modified to identify the location of pharmacodynamic characteristics more precisely. Secondly, the Distance Tolerance (DT) represents the distance between the pharmacodynamic characteristics. It can be added or subtracted based on the accuracy of the database search results to enhance reliability of pharmacophore model. Thirdly, the excluded volume (Ev) is an important form to constraint space, which generally produces a region of unfavorable atomic collisions by the different structure between active and inactive compounds. After adding the excluded volume, the predictive power and search capability of the model will be improved, correspondingly.

### 2.2. Database Screening

The optimal pharmacophore model with qualified evaluation indexes was utilized to screen CYP11B2 inhibitors from TCMD, which contains approximately 23033 natural compounds from 6735 medicinal plants [17]. The Flexible method was performed in the process of screening database. The hit compounds must be mapped with all the pharmacological features of the optimal model. The Fitvalue was a parameter to demonstrate the matching degree between compounds and the optimal pharmacophore model. Then, based on “Lipinski's rule of five” (≥4), molecular weight ≤ 500, H-bond donor ≤ 5, ALogP ≤ 5, and H-bond acceptor ≤ 10, the hit compounds were filtered to eliminate the nondrug-like compounds [18]. Finally, the compounds with Fitvalue higher than 0.80 and drug-like properties were selected as potential compounds for further analysis by molecular docking study.

### 2.3. Molecular Docking Studies

#### 2.3.1. Define Binding Site

The crystal structure of the human CYP11B2 was derived from the RCSB Protein Data Bank (PDB entry 4FDH, resolution 2.71 Å), complexed with an inhibitor of CYP11B2 named fadrozole and a coenzyme called iron porphyrin. During the preparing process of the protein, some common problems, such as nonstandard atom order in amino acids, incomplete residues, the existence of crystallographic waters, and the lack of hydrogen, were automatically cleaned up in 4FDH by DS [19]. The active pocket was created around the fadrozole using the Define and Edit Binding Site tools. Fadrozole was added to CHARMm force field and was assigned suitable protonation state at pH 8.5.

#### 2.3.2. Docking Strategy

After being removed from the crystal structure 4FDH, fadrozole was redocked into the crystal structure by using two common docking algorithms, LibDock and CDOCKER. The RMSD value was regarded as the index to determine the suitable docking module and check the rationality of the parameter settings of docking. The RMSD value is less than 2.00 Å, which suggests that the docking algorithm is reliable to reproduce the binding mode between initial compounds and protein [17]. The docking score of fadrozole was defined as threshold in identifying potential CYP11B2 inhibitors. Then, the compounds which mapped well with the optimal pharmacophore model and with drug-like property were docked into the active pocket in 4FDH. The potential compounds were selected on the basis of three indexes, Fitvalue higher than 0.80, docking score higher than threshold, and similar ligand-receptor interactions with initial compound. Finally, MD was carried out to verify the stability between the potential compounds and protein.

### 2.4. Molecular Dynamics Simulation

MD simulations were further implemented for the binding models of the potential CYP11B2 inhibitors using GROMACS 5.0.2 with the GROMOS96 43a1 force field [20]. The best binding mode of the potential compounds from the molecular docking results was implemented to MD. The GlycoBioChem PRODRG2 Server (http://davapc1.bioch.dundee.ac.uk/cgi-bin/prodrg) was used to generate the topology files of compounds. By using the simple point charge water model (SPC), the complex systems were solvated in a cubic box of a 10 Å dimension from the molecule periphery to cube edge. In order to neutralize the total charge of the systems, 5 chlorine ions were added randomly to replace water molecules. Then, each system was minimized by the steepest descent energy minimization until a Tolerance of 500 kJ·mol^−1^·nm^−1^.

To begin real dynamics, the equilibrations were conducted to equilibrate the solvent and ions around the protein. The system was gradually heating to target temperature of 300 K. A 50 ps NVT equilibration was run with position restraints at the constant temperature 300 K to cut down any bad contacts between protein and ligand [21]. Then, a 50 ps NPT equilibration was also conducted at 300 K with position restraints on protein backbone. The production of MD simulation was performed for 5 ns with a time-step of 0.5 fs using the v-rescale and Parrinello-Rahman method. The other parameters were set to default values. The system of fadrozole and protein was set as a reference and compared with potential inhibitors. The two evaluation criterions, the root mean-square deviation (RMSD) and total energy of the systems, were used to verify the stability of interactions between compounds and protein. And the change process of H-bond distances with time-evolution for potential inhibitors in the CYP11B2 active pocket was also analyzed, respectively.

## 3. Results and Discussion

### 3.1. Pharmacophore Model Generation

Ten pharmacophore hypotheses were generated based on twenty CYP11B2 active inhibitors in the training set (Table 1). Each pharmacophore model had A feature and H feature, which demonstrated that the two features played a crucial role for the activity of CYP11B2 inhibitors. All of the HipHop models had high rank scores, which indicated that compounds of the training set were well-mapped with generated pharmacophore models.

### 3.2. Validation and Optimization of the Pharmacophore Model

Ten pharmacophore model validation results from the test set were listed in Table 1. Furthermore, three assessment indexes, which were mentioned in Section 2.1.3, were used to select the best model. Unfortunately, all of the models were incompetent due to the fact that the achieved IEI and CAI values were all less than 2. Hence, one of the ten pharmacophore models which had the lowest ∑Ranking was optimized. The ordinal numbers, “first,” “second,” “third,” and so on, were designated to appraise all four evaluation indicators, which contain Rank score, HRA, IEI, and CAI. For instance, the Hypo1 with the highest Rank score was assigned as number 1, then Hypo 2 and Hypo 3 with the same score were assigned as number 2, Hypo 4 was assigned as number 3, and the remaining model might be deduced in this order. The results of ∑Ranking of ten models were detailed in Table 2.

**Figure 2:**
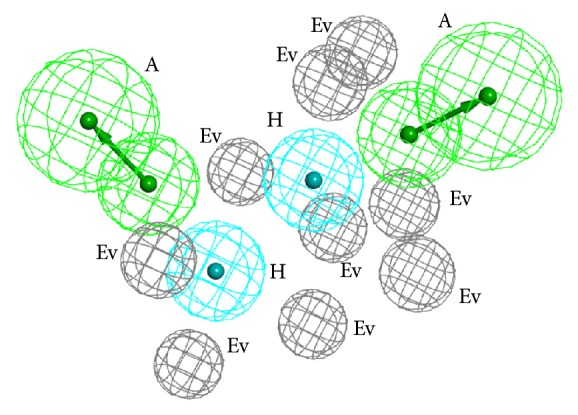
The optimal pharmacophore model Hypo9-1.

According to ranking results, the Hypo 9 with the lowest value of ∑Ranking was selected as initial pharmacophore model to be optimized. Based on the high active compounds and low active compounds in the training set, four excluded volumes were added to the Hypo 9. Fortunately, the obtained model achieved desirable evaluation results, which was named Hypo9-1. And the values of HRA, IEI, and CAI were 94.37%, 2.18, and 2.06, respectively. Then, to validate Hypo9-1, the compounds in the training set were mapped with the Hypo9-1 and 19 compounds were successfully mapped with all the pharmacological features. Therefore, the Hypo9-1 was selected as the optimal pharmacophore model to screen potential CYP11B2 inhibitors, and the optimal pharmacophore model was shown in Figure 2. Hypo9-1 contained two hydrogen bond acceptors, two hydrophobic features, and nine excluded volumes.

### 3.3. Database Search

Hypo9-1 pharmacophore model was used to screen CYP11B2 inhibitors from TCMD. Then a hit list of compounds was obtained with Best Flexible search option. Based upon Lipinski's rules, 1200 potential drug-likeness compounds were obtained. Finally, 405 drug-like compounds and the Fitvalue higher than 0.80 were selected to further docking study.

### 3.4. Molecular Docking

The active pocket was defined around fadrozole, and the sphere radius was 6.69 Å in 4FDH. The fadrozole was eliminated and then redocked into the crystal structure to calculate RMSD value for verifying the reliability of the two common docking algorithms, LibDock and CDOCKER. The results of RMSD values were 6.36 Å and 6.05 Å, respectively, which were all greater than 2.00 Å. In order to decrease the RMSD value, the size of radius of the active pocket was changed. When the sphere radius was 7.90 Å, RMSD value was 0.41 Å by using CDOCKER algorithm, but the LibDock algorithm which obtained the RMSD value was 6.34 Å. Therefore, CDOCKER algorithm was selected to perform molecular docking study. The -CDOCKER_ENERGY of fadrozole was 21.74, which was regarded as the threshold value in identifying potential CYP11B2 inhibitors.

**Figure 3:**
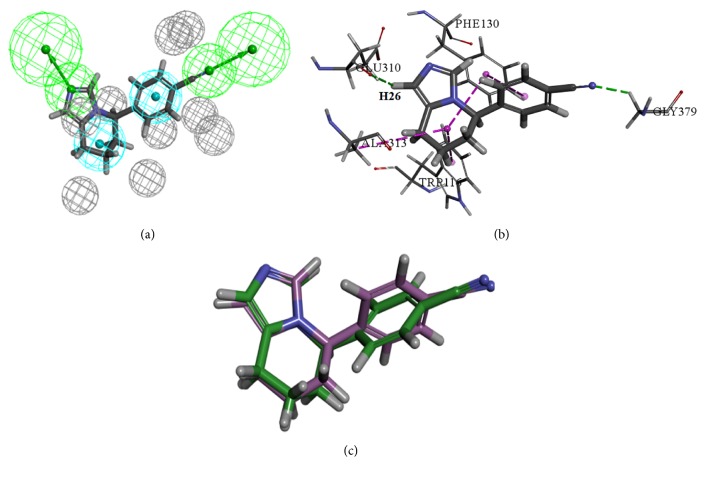
(a) The optimal pharmacophore model mapped with fadrozole. (b) The docking result of fadrozole. (c) Superimposition of the pharmacophore conformation (green) and docking conformation (purple).

The docking results revealed that the nitrogen atoms of triple bonds and H26 formed hydrogen bond interactions with amino acids GLY379 and GLU310, and they also mapped with two hydrogen bond acceptor features of the optimal pharmacophore model, respectively. Similarly, the benzene ring and carbon chain mapped with two hydrophobic features also formed hydrophobic interactions with PHE130, ALA313, and TRP116, respectively. The RMSD value of fadrozole between conformation of pharmacophore and conformation of docking was 0.68 which suggested that the results of the pharmacophore model and molecular docking were consistent. Thus the compounds which achieved the score greater than 21.74 and the interaction which was similar to fadrozole were selected as potential CYP11B2 inhibitors. The mapping result of fadrozole with optimal pharmacophore model, the interactions results between fadrozole and 4FDH, and the superimposed result of the conformation of fadrozole were shown in Figure 3.

**Figure 4:**
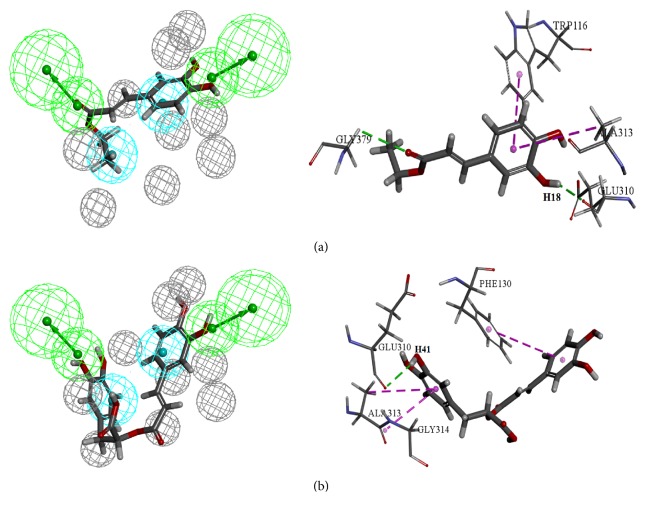
The mapping of the best pharmacophore model aligned with two potential compounds, ethyl caffeate (a) and labiatenic acid (b), as well as molecules interaction between two potential compounds and 4FDH.

The 405 drug-like compounds obtained from virtual screening were subjected to molecular docking employing CDOCKER program. Based on the two criterions, the docking score greater than 21.74, and the Fitvalue of pharmacophore higher than 0.80, a hit list of 145 compounds was obtained. Among the compounds, ethyl caffeate and labiatenic acid were two possible lead candidates in terms of the design structure of novel CYP11B2 inhibitors. The mapping results with the best pharmacophore model and the interactions between the two potential compounds and 4FDH were shown in Figures 4(a) and 4(b), respectively. For the ethyl caffeate (Figure 4(a)), the Fitvalue and docking score were 0.87 and 31.85, respectively. The two oxygen atoms of hydroxyl and ester group mapped with two hydrogen bond acceptor features and the oxygen atoms of carbonyl and H18 formed hydrogen bond interactions with GLY379 and GLU310, respectively. Furthermore, hydrophobic feature centered on the benzene ring, which formed the hydrophobic interactions with the amino acids ALA313 and TRP116. As shown in Figure 4(b), the Fitvalue and docking score of labiatenic acid were 0.88 and 54.00. The oxygen atoms of hydroxyl mapped with hydrogen bond acceptor features and H41 formed hydrogen bond interactions with GLU310. The two benzene rings mapped with two hydrophobic features also formed the hydrophobic interactions with PHE130 and ALA313, respectively. In a word, the binding mode of ethyl caffeate and labiatenic acid was similar to fadrozole.

### 3.5. Molecular Dynamics Simulation

**Figure 5:**
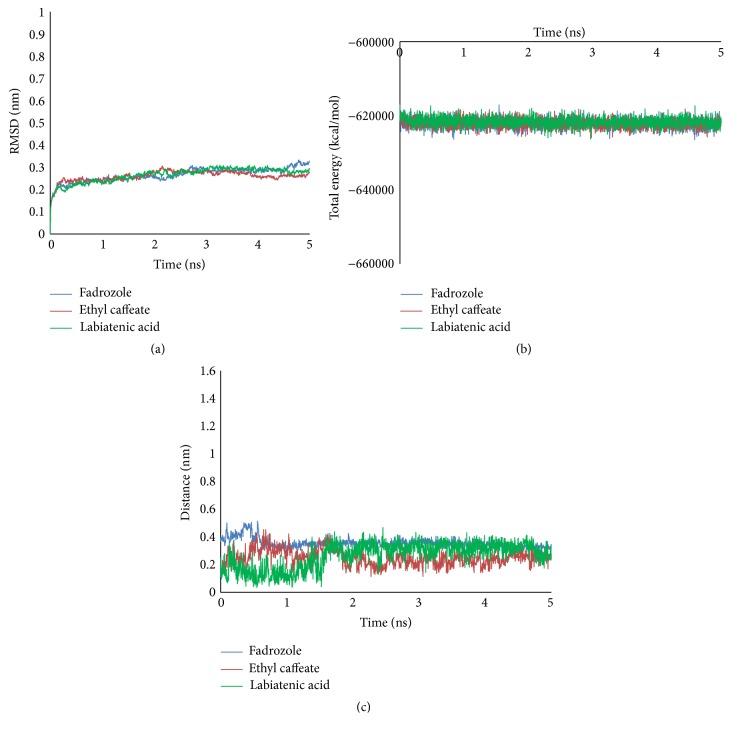
Trajectory of the MD simulation of fadrozole and two potential inhibitors. (a) Average backbone RMSD. (b) Total energy of complexes. (c) Distance between H of compound and O of GLU310. Blue indicates fadrozole, red indicates ethyl caffeate, and green indicates labiatenic acid.

MD was carried out to evaluate the stability of compounds and 4FDH under dynamic conditions. The best conformations of ethyl caffeate, labiatenic acid, and fadrozole obtained from docking results were used to run MD. The RMSD trajectories and total energy profiles of three complexes were shown in Figures 5(a) and 5(b). The results of RMSD indicated that three complexes approached to equilibrium phase in the 3 ns later, and the values converged to around 0.3 nm (Figure 5(a)). The total energy of two potential compounds was similar to fadrozole. The value was stably maintained in the systems, which approximated −620000 Kcal/mol (Figure 5(b)).

In addition, the hydrogen bond interaction is significant for the bioactivity of compounds. So the H-bond distances formed between three compounds and the binding pocket were calculated (Figure 5(c)). The result showed that the distances of H-bond reached stabilization after 3 ns. The H26 of fadrozole formed the hydrogen bond interaction with carboxyl oxygen atom of GLU310. The H18 of ethyl caffeate and H41 of labiatenic acid also formed hydrogen bond with carboxyl oxygen atom of GLU310, respectively. The result indicated that GLU310 might be the key site for inhibitory activity.

## 4. Conclusion

CYP11B2 plays a significant role in the biosynthesis of aldosterone which can cause a series of diseases, including hypertension. Therefore, discovering the novel CYP11B2 inhibitors had become a new approach for treatment hypertension. In this study, pharmacophore modeling, molecular docking, and MD were combined to discover novel CYP11B2 inhibitors from TCMD, which can ease the time-consuming and high-cost problems in drug research. The pharmacophore models were constructed by HipHop and the optimal model Hypo9-1 was obtained by adjusting the internal parameters. Then, the Hypo9-1 model was employed to screen potential CYP11B2 inhibitors from nature products. Based on the three criterions, the Fitvalue of pharmacophore higher than 0.80, the docking score greater than 21.74, and the interaction similar to fadrozole, two most promising CYP11B2 inhibitors, ethyl caffeate and labiatenic acid, were obtained. Finally, with further evaluation by MD, the interaction between the most promising compounds and protein was stable. In a word, based on all the experimental results, ethyl caffeate and labiatenic acid were regarded as the most potential CYP11B2 inhibitors and this study provided a new approach to discover novel CYP11B2 inhibitors from TCM.

## Figures and Tables

**Table 1:** The validation results of the pharmacophore models.

Hypo	Feature	Rank	*A*	*D*	Ha	Ht	HRA	IEI	CAI
1	RHHA Ev5	146.041	71	284	71	193	100.00%	1.47	1.47
2	RHHA Ev5	139.068	71	284	71	195	100.00%	1.46	1.46
3	RHHA Ev5	139.068	71	284	71	202	100.00%	1.41	1.41
4	HHAA Ev5	138.882	71	284	71	190	100.00%	1.50	1.50
5	HHAA Ev5	135.055	71	284	69	175	97.18%	1.58	1.53
6	RHHA Ev5	133.328	71	284	71	205	100.00%	1.39	1.39
7	RHHA Ev5	132.828	71	284	68	155	95.77%	1.75	1.68
8	RHHA Ev5	131.732	71	284	71	210	100.00%	1.35	1.35
9	HHAA Ev5	127.370	71	284	68	139	95.77%	1.96	1.87
10	RHHA Ev5	125.768	71	284	71	164	100.00%	1.73	1.73

*A* is the number of active compounds in the test set. *D* is the total number of compounds in test set. Ha is the number of active hits using pharmacophore to search. Ht is the number of hits using pharmacophore to search. HRA indicates the capability to recognize active molecules from the test set. IEI indicates the capability to recognize active molecules from nonactive molecules. CAI is the comprehensive appraisal index.

**Table 2:** The ranking results of ten models.

Hypo	Rank score^a^	HRA^a^	IEI^a^	CAI^a^	∑Ranking^b^
1	1	1	6	6	14
2	2	1	7	7	17
3	2	1	8	8	19
4	3	1	5	5	18
5	4	2	4	4	14
6	5	1	9	9	24
7	6	3	2	3	14
8	7	1	10	10	28
9	8	3	1	1	13
10	9	1	3	2	15

^a^The ranking results of the four indicators. ^b^The sum of the ranking values of the four indicators.
